# Deep Learning–Based Pattern Recognition for Detecting Penile Abnormalities: Protocol for Developing a Mobile App for Circumcision Eligibility

**DOI:** 10.2196/65811

**Published:** 2025-09-10

**Authors:** Irfan Wahyudi, Chandra Prasetyo Utomo, Samsuridjal Djauzi, Muhamad Fathurahman, Gerhard Reinaldi Situmorang, Arry Rodjani, Putu Angga Risky Raharja, Kevin Yonathan, Budi Santoso, Dwidian Khresna, Marco Raditya

**Affiliations:** 1 Department of Urology Faculty of Medicine Universitas Indonesia - Cipto Mangunkusumo Hospital Jakarta Indonesia; 2 YARSI E-Health Research Center Faculty of Information Technology YARSI University Jakarta Indonesia; 3 Department of Internal Medicine Faculty of Medicine Universitas Indonesia Jakarta Indonesia

**Keywords:** circumcision, artificial intelligence, penile abnormalities, mobile app, prospective cohort study

## Abstract

**Background:**

Circumcision is a widely practiced procedure with cultural and medical significance. However, certain penile abnormalities—such as hypospadias or webbed penis—may contraindicate the procedure and require specialized care. In low-resource settings, limited access to pediatric urologists often leads to missed or delayed diagnoses. Artificial intelligence (AI)–based image recognition presents a scalable solution to facilitate early detection and informed decision-making.

**Objective:**

This study aims to develop and validate an AI-powered image classification system integrated into a mobile app to detect penile abnormalities and assess circumcision eligibility. The system is designed to support preliminary screening by general practitioners and caregivers in underserved areas.

**Methods:**

A prospective cohort study was conducted involving pediatric patients at Cipto Mangunkusumo Hospital, Jakarta, Indonesia. Digital images will be collected prospectively from pediatric patients at Cipto Mangunkusumo Hospital captured by health care professionals or caregivers. High-resolution penile images were systematically captured from ventral, dorsal, and lateral angles and subsequently classified as either having normal or abnormal anatomy by urologists. Leveraging pretrained deep learning architectures, the AI models were developed to accurately classify these images and assess circumcision eligibility based on anatomical criteria. Image preprocessing included resizing, normalization, and augmentation. Transfer learning techniques were used to enhance accuracy. The model was developed using TensorFlow and Keras. Performance evaluation used accuracy, sensitivity, specificity, and *F*_1_-score. Following development, the model will be embedded into a mobile app to enable real-time analysis, with feedback on whether further clinical evaluation or referral is needed.

**Results:**

Model development began in January 2024 and is currently ongoing. Integration into the mobile app and deployment testing are scheduled across 3 sequential phases—refinement, integration, and user testing—through January 2026. Preliminary models have been trained, and refinement is underway to improve diagnostic accuracy and usability.

**Conclusions:**

The proposed AI-based system offers a promising tool to support early diagnosis of penile abnormalities and safe circumcision decision-making in resource-limited settings. Its integration into a mobile app enables preliminary screening outside specialized centers, facilitating telemedicine and optimizing referral pathways.

**International Registered Report Identifier (IRRID):**

DERR1-10.2196/65811

## Introduction

The surgical procedure of circumcision, involving the removal of the foreskin that covers the glans of the penis, is widely practiced around the world. This ancient practice holds substantial importance both medically and culturally [1]. Medically, circumcision is linked to several health benefits, including a lower risk of urinary tract infections, sexually transmitted infections, and penile cancer, as well as improved genital hygiene [2,3]. Culturally and religiously, circumcision is considered an essential ritual in various societies, making it a common procedure in many communities [4].

However, not every individual is a suitable candidate for circumcision. Contrary to popular belief, circumcision carries potential risks, particularly in the presence of certain penile abnormalities [1,5]. Conditions such as hypospadias, epispadias, buried penis, webbed penis, and ambiguous genitalia can contraindicate the procedure, necessitating more complex surgical interventions by specialized health care providers. The accurate identification of these anomalies is crucial to avoid complications and ensure optimal patient outcomes [6].

In many low-resource settings, the challenge of accurately diagnosing penile abnormalities is exacerbated by limited access to specialized health care providers. This can lead to delayed or missed diagnoses, resulting in suboptimal care and increased anxiety for families [7-11]. Advances in telemedicine and digital health technologies present promising solutions to these challenges by enhancing the accessibility and accuracy of medical assessments [12,13].

This research aims to develop a digital pattern recognition system using deep learning techniques to identify penile abnormalities and assess eligibility for circumcision. By integrating this system into a mobile app, we hope to provide a reliable and accessible tool for health care providers and families, ultimately improving the circumcision decision-making process and patient care.

## Methods

### Overview

This research uses a prospective cohort design to develop a sophisticated digital image recognition system designed to identify penile abnormalities. The study will enroll participants from Cipto Mangunkusumo Hospital in Jakarta, Indonesia, who meet specific inclusion criteria. Digital photographs of the penile region will be taken by health care professionals such as general practitioners or by the patients’ guardians, if necessary.

This protocol adheres to the guidelines outlined in the SPIRIT (Standard Protocol Items: Recommendations for Interventional Trials) 2013 checklist for reporting protocol studies [14]. During execution of the protocol, any modifications made by the lead researcher will be reported in the subsequent publications.

Participants will be selected using a consecutive sampling method until a sufficient number of images are obtained to ensure robust training and validation of the artificial intelligence (AI) model. The inclusion criteria for this study require participants to be male patients younger than 18 years who have not undergone prior circumcision or penile surgery. Exclusion criteria include cases where adequate image quality cannot be obtained due to medical or technical constraints, as well as situations in which the patient’s guardians do not provide consent for data collection or participation. These criteria ensure that the study includes a relevant patient population while maintaining ethical and methodological integrity.

Digital images will be captured from 3 angles—ventral, dorsal, and lateral—to provide a comprehensive view of the penile anatomy. Data collection will be performed upon agreement to participate by the patients’ guardian by the attending physician.

**Figure 1:**
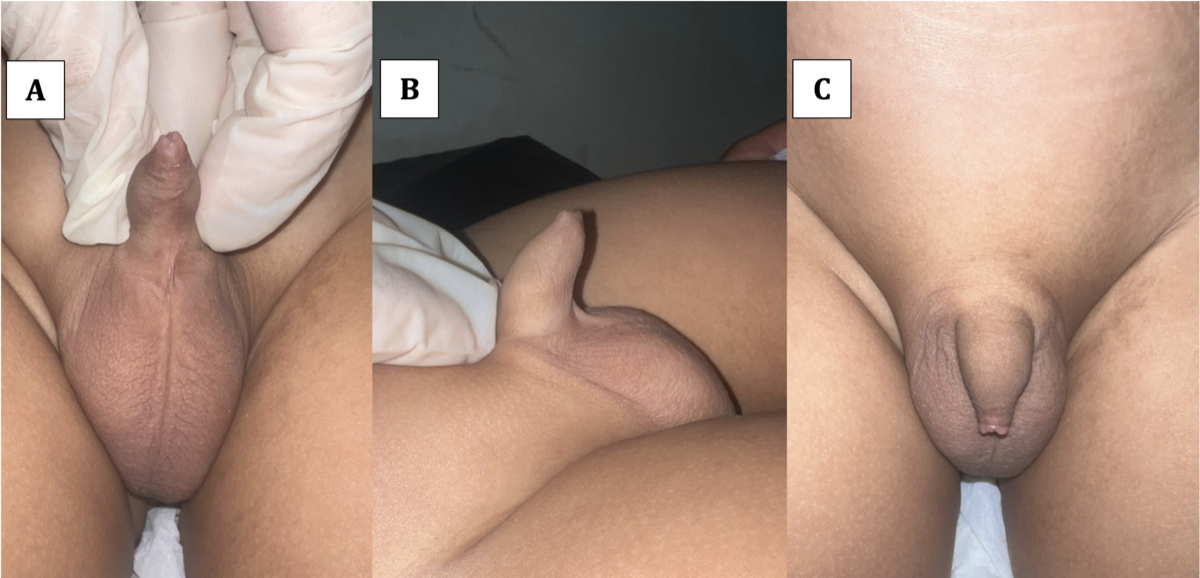
Example of photographs taken for the (A) ventral aspect, (B) lateral aspect, and (C) dorsal aspect of the penis.

Prior to capturing penile images, both general practitioners and the patient’s guardians will be provided with a standardized reference illustration. This visual guide aims to minimize variations in camera angles across different perspectives, ensuring consistency in image acquisition. The standardization process is informed by established methodologies from previous research [15]. Examples of photographs of the penis structure are shown in Figure 1.

The collected images will undergo preprocessing to standardize dimensions and ensure uniformity. These images will be categorized according to the angle of the image taken. Duplicate images will be removed to avoid bias, and images will be resized to a standard dimension suitable for deep learning models. The annotated images will be categorized into 2 groups: those showing normal penile anatomy and those depicting abnormalities.

The AI model will be developed using advanced deep learning architectures including VGG16 [16], MobileNet [17], and DenseNet121 [18], selected for their effectiveness in medical image classification. The initial dataset will be split into training, validation, and testing subsets to facilitate model development and optimization. Data augmentation techniques will be used to enhance the training dataset by artificially increasing its size and diversity [19].

The AI models will be trained using the TensorFlow framework [20] and the Keras library [21], which offer streamlined processes for constructing, training, and validating deep learning models. Transfer learning will be used to overcome the challenges posed by a limited dataset, leveraging pretrained models to improve accuracy. Digital images from the collected dataset will be randomly categorized into training, validation, and testing data by a team of authors dedicated to developing the AI model. An external dataset will not be used in order to conform uniformity in the format of image taken.

Performance evaluation will be conducted using standard classification metrics, including accuracy, sensitivity, specificity, and *F*_1_-score. AI-generated results will be compared against the clinical diagnoses documented in patients’ medical records based on their physical examinations. Additionally, the functionality and usability of the mobile app will be assessed through user feedback, which will be collected and analyzed to identify areas for improvement. A web-based questionnaire developed by the authors will be distributed to urologists recruited to use the app. The questionnaire will consist of both open-ended questions and Likert scale items designed to assess user experience with the AI model and the mobile app. Participants will be recruited through web-based invitations sent to local urologists.

Upon completion of model development, the AI system will be integrated into a novel mobile app. This app will allow health care providers and guardians to capture and upload penile images, which will then be analyzed in real-time by the AI model. The system will provide immediate feedback on the presence of penile abnormalities, aiding in the decision-making process for circumcision eligibility.

### Ethical Considerations

Ethical guidelines, as per the Declaration of Helsinki, will be strictly followed. The study protocol has received ethical approval from the Medical Research Ethics Committee at Universitas Indonesia (22-03-0310). Informed consent will be obtained from the guardians of all participants by the research team. Collected images will be anonymized and securely stored, ensuring privacy and confidentiality. Research databases will only be made accessible to authors. The identities of patients will not be revealed to AI model developers. Patients’ identities are only kept by the physician performing the corresponding physical examination and capturing the photograph.

## Results

The enrolment of study participants commenced in January 2023, while the development of the digital recognition system and mobile app began in January 2024. With adherence to this protocol, the study’s findings are anticipated to be finalized by January 2026 and will be shared via publications.

Currently, the study has successfully pooled a total of 633 penis images, comprising 338 normal and 295 abnormal penis images. These images have been successfully integrated into 3 pretrained AI models. Although the current results are promising, we aim to further improve the model’s accuracy before clinical implementation to ensure greater reliability and robustness. Thus, the next stage of the project involves both AI model refinement and AI model development testing. In this phase, we aim to further enhance the model’s accuracy by expanding the dataset and experimenting with integrating various pretrained AI models. This phase is expected to take approximately 6 months, depending on the complexity of dataset expansion and model integration.

Simultaneously, we will initiate the model integration phase, embedding the AI model into our mobile app to evaluate its performance within the product environment. This phase is projected to last 3 months, allowing time for troubleshooting, bug fixes, and initial user testing within the app.

Upon successful integration, the project will progress to the model deployment testing phase, which will include comprehensive user testing to gather valuable feedback. This phase is expected to take 3 months to allow adequate time for user feedback, analysis, and necessary adjustments based on the findings. This feedback will guide our team in fine-tuning model integration and optimizing user experience, ensuring the product meets practical and clinical needs. In light of this, the project is expected to be completed by January 2026.

## Discussion

### Principal Findings

This study introduces an AI-driven digital pattern recognition system designed to detect penile abnormalities and assess circumcision eligibility. By leveraging deep learning methodologies and mobile technology, this research aims to enhance diagnostic accuracy and accessibility, particularly in resource-constrained settings [13,22]. The proposed system enables real-time analysis, providing immediate clinical decision support for health care professionals and guardians [23]. By addressing the limitations of traditional diagnostic approaches, this study has the potential to reduce missed or delayed diagnoses, thereby improving patient outcomes and health care efficiency [7,9]. In practice, these findings suggest that AI-assisted real-time examination could help screen patients before circumcision. If no penile abnormalities are detected, the patient would be deemed eligible and could safely proceed with the procedure.

### Comparison With Prior Work

Existing literature has demonstrated the efficacy of AI in medical imaging across multiple disciplines, including dermatology, radiology, and ophthalmology [19]. However, research on AI applications within urology, particularly in evaluating circumcision eligibility, remains scarce [15,24]. Prior studies on digital health solutions for genital abnormalities have predominantly focused on telemedicine-based consultations rather than automated diagnostic tools [22,25]. This study builds upon previous advancements by introducing a novel, AI-based approach tailored specifically for penile abnormality detection [8,9]. Unlike conventional clinical evaluations that necessitate specialized expertise, the proposed system offers a scalable, cost-effective alternative that enhances diagnostic capabilities in low-resource settings [11,26].

### Strengths and Limitations

A key strength of this study lies in its innovative application of AI for detecting penile abnormalities and assessing circumcision eligibility [13]. By integrating a digital image recognition system into a mobile platform, the proposed solution facilitates real-time decision-making and broadens access to specialized care [22,23]. Additionally, the study uses a structured methodological framework, incorporating robust data collection, preprocessing, and validation techniques to ensure model reliability and accuracy [19,27].

Despite these strengths, the study has certain limitations. The reliance on digital images captured by nonspecialists (ie, guardians) may introduce inconsistencies in image quality, potentially affecting the AI model’s performance [7,9,26]. Furthermore, while data augmentation techniques are used, the dataset size remains a limitation, as deep learning models typically require large, diverse datasets to achieve optimal generalizability [19,28,29]. Ethical considerations related to image collection, data security, and patient confidentiality also pose challenges that must be meticulously addressed through stringent privacy measures and adherence to ethical research standards [14,23,27]. Future research should prioritize dataset expansion, enhanced image standardization techniques, and continuous validation to mitigate these limitations and improve model robustness [25,28,30].

### Future Directions

To further refine the AI system, future research should explore advanced deep learning architectures and optimization strategies that enhance model accuracy and efficiency [13,19,29]. Expanding the dataset to encompass a wider demographic range will improve the system’s generalizability and reliability [9-11]. Additionally, implementing an interactive feedback loop within the mobile app by enabling health care providers to verify and refine AI-generated diagnoses could strengthen its clinical applicability [7,10,11]. Investigating the integration of AI with complementary diagnostic modalities, such as ultrasound imaging, may further enhance the system’s diagnostic precision [13,25,27]. Longitudinal studies evaluating real-world implementation and user experience will be crucial in ensuring the system’s efficacy and scalability [22,23,30].

### Dissemination Plan

The findings of this study will be disseminated through high-indexed Scopus, peer-reviewed journal publications and international conference presentations to engage the academic and medical communities. Collaboration with health care institutions, policymakers, and professional organizations will facilitate the implementation of the AI-based system in clinical practice [4,24]. Additionally, integrating AI-assisted decision-making tools into existing clinical workflows will be explored to enhance accessibility and user adoption among health care providers. By leveraging multidisciplinary partnerships and outreach strategies, this research aims to contribute to the broader advancement of AI-driven telemedicine and digital health interventions, ultimately improving patient care and clinical decision-making processes.

## Appendix

Multimedia Appendix 1 User acceptance test questionnaire for the mobile app.

Multimedia Appendix 2 Peer-Review Report by Kementrian Riset Dan Teknologi/Badan Riset Dan Inovasi Nasional Deputi Bidang Penguatan Riset Dan Pengembangan (RISTEK-BRIN; Ministry of Research and Technology/National Research and Innovation Agency - Deputy for Strengthening Research and Development) (Jakarta, Indonesia).

Multimedia Appendix 3 SPIRIT checklist.

## Abbreviations

AI: artificial intelligence
SPIRIT: Standard Protocol Items: Recommendations for Interventional Trials

## Data Availability

The datasets generated and analyzed during the current study are not publicly available due to ethical and privacy restrictions, as they contain identifiable clinical images of pediatric patients. Access to anonymized data may be made available from the corresponding author upon reasonable request and with approval from the Medical Research Ethics Committee of Universitas Indonesia, in accordance with institutional and national data protection regulations.
