# Correction: Deep Learning–Based Pattern Recognition for Detecting Penile Abnormalities: Protocol for Developing a Mobile App for Circumcision Eligibility

**DOI:** 10.2196/89351

**Published:** 2026-03-02

**Authors:** Irfan Wahyudi, Chandra Prasetyo Utomo, Samsuridjal Djauzi, Muhamad Fathurahman, Gerhard Reinaldi Situmorang, Arry Rodjani, Putu Angga Risky Raharja, Kevin Yonathan, Budi Santoso, Dwidian Khresna, Marco Raditya

**Affiliations:** 1 Department of Urology Faculty of Medicine Universitas Indonesia - Cipto Mangunkusumo Hospital Jakarta Indonesia; 2 YARSI E-Health Research Center Faculty of Information Technology YARSI University Jakarta Indonesia; 3 Department of Internal Medicine Faculty of Medicine Universitas Indonesia Jakarta Indonesia

In “Deep Learning–Based Pattern Recognition for Detecting Penile Abnormalities: Protocol for Developing a Mobile App for Circumcision Eligibility” [1], the authors noted an error in the funding information in the Acknowledgments section of the published version. The correction has been made to ensure accurate attribution of grant sources and contract numbers.

In the originally published article, the Acknowledgments section read:

We would like to acknowledge the support of the Badan Riset dan Inovasi Nasional for their financial support to fund this research (grant NKB-912/UN2.RST/HKP.05.00/2022). We would also like to extend our sincere appreciation to the entire medical team for their vital contributions in executing this protocol and gathering the research data. Huge appreciation is also given to the staff members of the E-Health Research Center for their help in providing support throughout the AI development process.

The corrected Acknowledgments section now reads:

*This study was funded by the RIIM LPDP Grant Program and Badan Riset dan Inovasi Nasional (BRIN; grants 97/IV/KS/11/2022 and 530./PKS/WRIII-DISTP/UI/2022), awarded through Universitas Indonesia. We would also like to extend our sincere appreciation to the entire medical team for their vital contributions in executing this protocol and gathering the research data. Huge appreciation is also given to the staff members of the YARSI E-Health Research Center for their help in providing support throughout the AI development process.*

The correction will appear in the online version of the paper on the JMIR Publications website, together with the publication of this correction notice. Because this was made after submission to PubMed, PubMed Central, and other full-text repositories, the corrected article has also been resubmitted to those repositories.
