# Supplementary material for: Deep Learning–Based Pattern Recognition for Detecting Penile Abnormalities: Protocol for Developing a Mobile App for Circumcision Eligibility
Source: JMIR Res Protoc. 2025 Sep 10;14:e65811. doi: 10.2196/65811 (PMC12461175; doi:10.2196/65811)
Supplement: Multimedia Appendix 1 [file resprot_v14i1e65811_app1.docx]

**User Acceptance Test Questionnaire for ASHOKA**

Thank you for participating in the User Acceptance Test (UAT) of our mobile application, ASHOKA. Your feedback is very important and will help us improve the app before launch. Please read each question carefully and answer honestly based on your experience using the app.

For questions with a **rating scale**, circle **one number** that best reflects your level of satisfaction or ease of use, where:

***1****= Very Unsatisfied / Difficult*

***2****= Unsatisfied / Somewhat Difficult*

***3****= Neutral / Acceptable*

***4****= Satisfied / Easy*

***5****= Very Satisfied / Very Easy*

**Name :**

**Institution :**

**Functionality**

1. The app correctly guides you through the process of taking the necessary photos (side, underside, topside)

**1** **2** **3** **4** **5**

1. You are able to capture or upload photos easily.

**1** **2** **3** **4** **5**

1. Photo editing tools (crop, rotate, discard) work as expected.

**1** **2** **3** **4** **5**

1. AI model able to successfully receive and process the uploaded images?

**1** **2** **3** **4** **5**

1. The final result screen clearly displays the AI's determination of normal or abnormal.

**1** **2** **3** **4** **5**

**Usability**

1. How intuitive did you find the navigation within the AI feature?

**1** **2** **3** **4** **5**

1. Were the instructions for taking and uploading photos clear and easy to follow?

**1** **2** **3** **4** **5**

1. Did you encounter any difficulties in using the photo editing functions?

**1** **2** **3** **4** **5**

1. How would you rate the clarity of the results presented to you?

**1** **2** **3** **4** **5**

1. Were you able to understand the probabilities associated with each classification?

**1** **2** **3** **4** **5**

**Performance**

1. How was the response time when taking/uploading photos?

**1** **2** **3** **4** **5**

1. Was there any noticeable lag or delay in processing images through the AI model?

**1** **2** **3** **4** **5**

1. How long did it take to receive the classification results after image submission?

**1** **2** **3** **4** **5**

**Security and Privacy**

1. Were you provided with information about data security for your photos?

**1** **2** **3** **4** **5**

1. Do you feel confident that your personal information is secure with the ASHOKA app?

**1** **2** **3** **4** **5**

1. Was the purpose and the handling of the photos clearly explained before you started using the application?

**1** **2** **3** **4** **5**

**Overall Satisfaction**

1. How satisfied are you with the overall workflow of the AI feature?

**1** **2** **3** **4** **5**

1. Is there anything about the process that you felt could be improved?
   ________________________________________________________________________________________________________________________________________________________________________________________________________________________________________________________________________________________________________________________________________________________________________
