# Supplementary material for: Deep Learning–Based Pattern Recognition for Detecting Penile Abnormalities: Protocol for Developing a Mobile App for Circumcision Eligibility
Source: JMIR Res Protoc. 2025 Sep 10;14:e65811. doi: 10.2196/65811 (PMC12461175; doi:10.2196/65811)
Supplement: Multimedia Appendix 2 [file resprot_v14i1e65811_app2.pdf]

KEMENTRIAN RISET DAN TEKNOLOGI /
BADAN RISET DAN INOVASI NASIONAL
DEPUTI BIDANG PENGUATAN RISET DAN PENGEMBANGAN
Gedung BJ Habibie Lantai 19 – 20, Jalan M.H. Thamrin Nomor 8, Jakarta 10340
Telepon: (021) 3169707; Faksimile: (021) 3101728, 3102368
Laman: www.risbang.ristekbrin.go.id

Reviewers' Comments for Author's Rebuttal

Manuscript titled "Digital Pattern Recognition Using Deep Learning Architectures to Detect Penile Abnormalities: Protocol for the Development of a Mobile Application for Circumcision Eligibility"

Please write down your response or revision in **"Author's Response / Revision"** column. Locate the changes you've made or the sentence that you refer to as their page and line in **"Location in Text"** column. Please also mark the changes you have made **in your manuscript** by highlighting or changing the font color.

Reviewer

No.	Aspect	Comments	Author's Response / Revision	Location in Text
	Title	**REVIEWER 1**<br>The title has described the paper appropriately<br><br>**REVIEWER 2**<br>The title is too lengthy. Consider shortening it while retaining the key information, maybe removing the "for the Development of a Mobile Application."	**REVIEWER 1**<br>Thank you for your comment<br><br>**REVIEWER 2**<br>We believe the phrase "for the Development of a Mobile Application" is essential to convey the full scope of the study, so we have opted to retain it. However, we have made the title more concise by revising other parts.	Title

	Abstract	**REVIEWER 1**<br>The abstract is generally well-structured but consider adding a sentence about the potential implications for clinical practice.<br><br>**REVIEWER 2**<br>The background is too long.	**REVIEWER 1:**<br>We have added a sentence to the abstract highlighting the potential implications for clinical practice, particularly in enhancing decision-making regarding circumcision<br><br>**REVIEWER 2:**<br>We have condensed the background information in the abstract	Abstract
	Introduction	**REVIEWER 1**<br>The introduction is thorough, but consider adding details on how this could benefit a low-resource setting<br><br>**REVIEWER 2**<br>The introduction is already sufficient	**REVIEWER 1:**<br>We have added details on this matter in the third paragraph<br><br>**REVIEWER 2:**<br>Thank you for your comment	Introduction
	Methods	**REVIEWER 1**<br>The method section is easy to understand, but consider adding more details on which deep learning algorithms will be used along with the reason.<br><br>**REVIEWER 2**<br>The method is clear. However, please add image examples for the angles for the digital image to provide more clarity	**REVIEWER 1:**<br>We have added sentences in the "Development of AI Model" section, providing examples.<br><br>**REVIEWER 2:**<br>We have added examples of the photograph in the "Data Collection" section. The patient has consented to this	Methods

			image being used for publication.	
	Results	**REVIEWER 1**<br>The result is appropriate<br><br>**REVIEWER 2**<br>Provide an estimation on when the study might be concluded	**REVIEWER 1:**<br>Thank you for your comment<br><br>**REVIEWER 2:**<br>We have added additional information regarding our estimation on when the study will be concluded	Results
	Discussion	**REVIEWER 1**<br>The discussion has thoroughly described the potential benefits along with the feasibility of this project, but consider adding more details on why the authors opted for developing AI in a mobile application. Also, consider adding the possibility of having limited sample size as a limitation, as congenital genital defects might not be easy to come by.<br><br>**REVIEWER 2**<br>The discussion is clear. However, it would be better to add previous studies supporting whether telemedicine is applicable in Indonesia.	**REVIEWER 1:**<br>We have added explanation on why we opted for AI in a mobile application within the fourth paragraph. We have also revised the fifth paragraph accordingly, adding a limited sample size as a potential limitation.<br><br>**REVIEWER 2:**<br>We have added explanation on this matter in the first paragraph of the discussion section.	Discussion
